# *rac*-Ammonium *cis*-2-carb­oxy­cyclo­hexane-1-carboxyl­ate

**DOI:** 10.1107/S1600536810051883

**Published:** 2010-12-18

**Authors:** Graham Smith, Urs D. Wermuth

**Affiliations:** a Faculty of Science and Technology, Queensland University of Technology, GPO Box 2434, Brisbane, Queensland 4001, Australia

## Abstract

In the structure of the title compound, NH_4_^+^·C_8_H_11_O_4_^−^, the carboxyl and carboxyl­ate groups of the cation adopt C—C—C—O torsion angles of 174.9 (2) and −145.4 (2)°, respectively, with the alicyclic ring. The ammonium H atoms of the cations give a total of five hydrogen-bonding associations with carboxyl­ate O-atom acceptors of the anion which, together with a carboxyl O—H⋯O_carboxyl­ate_ inter­action give sheet structures which lie in the (101) planes.

## Related literature

For the structure of the isomeric racemic ammonium salt of *trans*-cyclo­hexane-1,2-dicarb­oxy­lic acid (TCDA), see: Stibrany *et al.* (2004 ▶). For the structures of *rac-cis*-CDA, *rac-trans*-CDA and (+)-*trans*-CDA, see: Benedetti *et al.* (1970 ▶); Benedetti, Corradini, Pedone & Post (1969 ▶); Benedetti, Corradini & Pedone (1969 ▶); Rizal & Ng (2008 ▶). The *cis,trans*-isomer exists as an essentially unresolvable racemate, see: Eliel (1962 ▶). For hydrogen-bond motifs, see: Etter *et al.* (1990 ▶). 
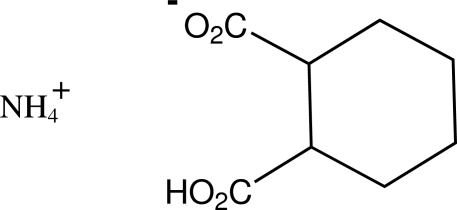


## Experimental

### 

#### Crystal data

NH_4_^+^·C_8_H_11_O_4_^−^
*M* *_r_* = 189.21
Monoclinic, 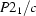

*a* = 15.4908 (13) Å
*b* = 5.3475 (3) Å
*c* = 12.1716 (9) Å
β = 109.795 (9)°
*V* = 948.68 (13) Å^3^
*Z* = 4
Mo *K*α radiation
μ = 0.11 mm^−1^
*T* = 200 K
0.30 × 0.22 × 0.10 mm

#### Data collection

Oxford Diffraction Gemini-S CCD-detector diffractometer
Absorption correction: multi-scan (*CrysAlis PRO*; Oxford Diffraction, 2010 ▶) *T*_min_ = 0.86, *T*_max_ = 0.98
5997 measured reflections
1862 independent reflections
1313 reflections with *I* > 2σ(*I*)
*R*_int_ = 0.046

#### Refinement

*R*[*F* ^2^ > 2σ(*F* ^2^)] = 0.058
*wR*(*F* ^2^) = 0.141
*S* = 0.99
1862 reflections
138 parameters
H atoms treated by a mixture of independent and constrained refinement
Δρ_max_ = 0.44 e Å^−3^
Δρ_min_ = −0.22 e Å^−3^

### 

Data collection: *CrysAlis PRO* (Oxford Diffraction, 2010 ▶); cell refinement: *CrysAlis PRO*; data reduction: *CrysAlis PRO*; program(s) used to solve structure: *SHELXS97* (Sheldrick, 2008 ▶); program(s) used to refine structure: *SHELXL97* (Sheldrick, 2008 ▶); molecular graphics: *PLATON* (Spek, 2009 ▶); software used to prepare material for publication: *PLATON*.

## Supplementary Material

Crystal structure: contains datablocks global, I. DOI: 10.1107/S1600536810051883/ng5083sup1.cif

Structure factors: contains datablocks I. DOI: 10.1107/S1600536810051883/ng5083Isup2.hkl

Additional supplementary materials:  crystallographic information; 3D view; checkCIF report

## Figures and Tables

**Table 1:** Hydrogen-bond geometry (Å, °)

*D*—H⋯*A*	*D*—H	H⋯*A*	*D*⋯*A*	*D*—H⋯*A*
N1—H1*A*⋯O11	0.90 (3)	2.22 (3)	3.012 (3)	146 (3)
N1—H1*A*⋯O12	0.90 (3)	2.44 (3)	3.237 (3)	147 (3)
N1—H1*B*⋯O12^i^	0.91 (4)	1.96 (4)	2.835 (3)	161 (4)
N1—H1*C*⋯O11^ii^	0.97 (2)	1.85 (3)	2.811 (3)	168 (2)
N1—H1*D*⋯O12^iii^	0.99 (3)	1.86 (3)	2.842 (3)	174 (3)
O22—H22⋯O11^iv^	0.88 (4)	1.76 (4)	2.619 (3)	165 (5)

Symmetry codes: (i) 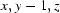; (ii) 
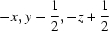
; (iii) 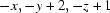; (iv) 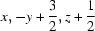.

**Fig. 1.**
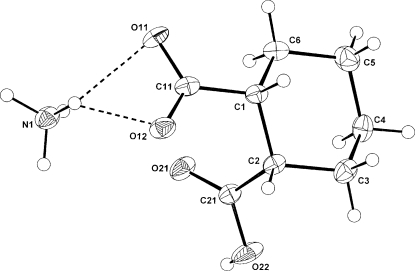
Molecular configuration and atom naming scheme for the ammonium cation the CDA anion in (I). Inter-species hydrogen bonds are shown as dashed lines and displacement ellipsoids are drawn at the 40% probability level.

**Fig. 2.**
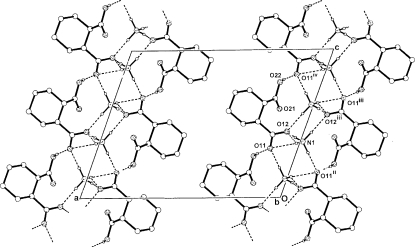
The two-dimensional hydrogen-bonded sheet structures in (I) which extend down the (101) planes in the unit cell, showing hydrogen-bonding interactions as dashed lines. Non-associative H atoms are omitted. For symmetry codes, see Table 1.

**Fig. 3.**
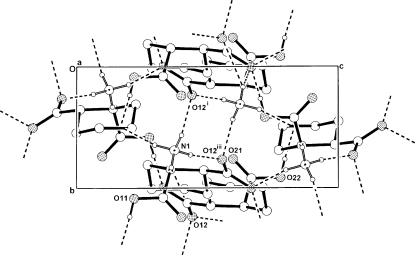
A portion of the sheet structure in (I) viewed down the a axis of the unit cell.

## supplementary crystallographic information

### Comment

Cyclohexane-1,2-dicarboxylic acid (CDA) is of interest conformationally since the *cis,cis*- (or *trans,trans*)- configurational isomers (the *trans* form) may be resolved while the *cis,trans*-isomer exists as an essentially unresolvable racemate (Eliel, 1962). The structures of both racemic-*trans*-CDA (TCDA) (Benedetti, Corradini, Pedrone & Post, 1969; Rizal & Ng, 2008), and (+)-*trans*-CDA (Benedetti, Corradini, Pedrone & Post, 1969) are known as well as that of racemic-*cis*-CDA (CCDA) (Benedetti *et al.*, 1970). Our reaction of cyclohexane-1,2-dicarboxylic anhydride in 50% ethanol/water with an ammoniacal solution gave, after evaporation, crystals which were found to have a monoclinic unit cell which was very similar to that previously reported for the roon-temperature structure of ammonium *trans*-2-carboxycyclohexanecarboxylate (Stibrany *et al.*, 2004) [*a* = 15.712 (7), *b* = 6.141 (3), *c* = 10.464 (5) Å, β = 104.96 (4)°, *V* = 975.5 (8) Å^3^, *Z* = 4, space group *P*2_1_/*c*], suggesting either a crystal polymorph or the configurational *cis*-isomeric salt. The compound has been confirmed as the racemic *cis*-salt of CDA, NH_4_^+^ C_8_H_11_O_4_^-^ (I) and the structure is reported here.

With (I) (Fig. 1) the ammonium cations give five hydrogen-bonding interactions with carboxylate O-atom acceptors of the anion (Table 1), including a three-centre asymmetric cyclic N—H···O,O' association [graph set R^2^_1_(4) (Etter *et al.*, (1990)]. The two-dimensional sheet structures generated extend along the (101) planes in the unit cell (Fig. 2) with the ammonium ions lying close to these planes and providing the linkages within the sheets (Fig. 3), together with strong carboxylic acid O—H···O_carboxyl_ hydrogen bonds. This and all other features of the hydrogen bonding in (I), including the centrosymmetric cyclic R^2^_4_(8) heteromolecular motifs, are similar to those of the *trans*-CDA ammonium salt (Stibrany *et al.*, 2004) but conformationally, the anions differ although not in a major way. Comparative carboxylic acid and carboxylate groups defined by torsion angles C1–C2–C21–O22 [174.9 (2)°] and C2–C1–C11–O11 [-145.4 (2)°] in (I) compare with -166.66 (19) and 137.3 (2)° respectively for the *trans* salt but are more comparable with -178.8 (5) and 152.9 (2)° for the *rac-cis*-CDA acid (Benedetti *et al.*, 1970).

### Experimental

The title compound was synthesized by reacting 1 mmol of cyclohexane-1,2-dicarboxylic anhydride with 50 ml of an 5*M* ammoniacal 1:1 ethanol–water solution. The solution was allowed evaporate to moist dryness at room temperature over several months, finally giving colourless poorly formed plates of (I) from which a specimen was cleaved for the X-ray analysis.

### Refinement

Hydrogen atoms involved in hydrogen-bonding interactions were located by difference methods and their positional and isotropic displacement parameters were refined. Other H-atoms were included in the refinement at calculated positions [C–H = 0.96–0.97 Å and with *U*_iso_(H) = 1.2*U*_eq_(C), using a riding-model approximation.

### Crystal data

**Table:**

NH_4_^+^·C_8_H_11_O_4_^−^	*F*(000) = 408
*M**_r_* = 189.21	*D*_x_ = 1.325 Mg m^−^^3^
Monoclinic, *P*2_1_/*c*	Mo *K*α radiation, λ = 0.71073 Å
Hall symbol: -P 2ybc	Cell parameters from 2294 reflections
*a* = 15.4908 (13) Å	θ = 3.4–28.6°
*b* = 5.3475 (3) Å	µ = 0.11 mm^−^^1^
*c* = 12.1716 (9) Å	*T* = 200 K
β = 109.795 (9)°	Plate, colourless
*V* = 948.68 (13) Å^3^	0.30 × 0.22 × 0.10 mm
*Z* = 4	

### Data collection

**Table:**

Oxford Diffraction Gemini-S CCD-detector diffractometer	1862 independent reflections
Radiation source: Enhance (Mo) X-ray source	1313 reflections with *I* > 2σ(*I*)
graphite	*R*_int_ = 0.046
Detector resolution: 16.077 pixels mm^-1^	θ_max_ = 26.0°, θ_min_ = 3.4°
ω scans	*h* = −19→12
Absorption correction: multi-scan (*CrysAlis PRO*; Oxford Diffraction, 2010)	*k* = −6→6
*T*_min_ = 0.86, *T*_max_ = 0.98	*l* = −15→15
5997 measured reflections	

### Refinement

**Table:**

Refinement on *F*^2^	Primary atom site location: structure-invariant direct methods
Least-squares matrix: full	Secondary atom site location: difference Fourier map
*R*[*F*^2^ > 2σ(*F*^2^)] = 0.058	Hydrogen site location: inferred from neighbouring sites
*wR*(*F*^2^) = 0.141	H atoms treated by a mixture of independent and constrained refinement
*S* = 0.99	*w* = 1/[σ^2^(*F*_o_^2^) + (0.0838*P*)^2^] where *P* = (*F*_o_^2^ + 2*F*_c_^2^)/3
1862 reflections	(Δ/σ)_max_ < 0.001
138 parameters	Δρ_max_ = 0.44 e Å^−^^3^
0 restraints	Δρ_min_ = −0.22 e Å^−^^3^

### Special details

**Table:**

Geometry. Bond distances, angles *etc*. have been calculated using the rounded fractional coordinates. All su's are estimated from the variances of the (full) variance-covariance matrix. The cell e.s.d.'s are taken into account in the estimation of distances, angles and torsion angles
Refinement. Refinement of *F*^2^ against ALL reflections. The weighted *R*-factor *wR* and goodness of fit *S* are based on *F*^2^, conventional *R*-factors *R* are based on *F*, with *F* set to zero for negative *F*^2^. The threshold expression of *F*^2^ > σ(*F*^2^) is used only for calculating *R*-factors(gt) *etc*. and is not relevant to the choice of reflections for refinement. *R*-factors based on *F*^2^ are statistically about twice as large as those based on *F*, and *R*- factors based on ALL data will be even larger.

### Fractional atomic coordinates and isotropic or equivalent isotropic displacement parameters (Å^2^)

**Table:**

	*x*	*y*	*z*	*U*_iso_*/*U*_eq_	
O11	0.13732 (12)	0.9964 (3)	0.33214 (13)	0.0306 (5)	
O12	0.08448 (12)	1.2330 (3)	0.44276 (14)	0.0329 (6)	
O21	0.16061 (13)	0.7624 (3)	0.59854 (14)	0.0368 (6)	
O22	0.20622 (15)	0.9149 (4)	0.77916 (15)	0.0450 (7)	
C1	0.24584 (16)	1.1450 (4)	0.51309 (18)	0.0243 (7)	
C2	0.24599 (17)	1.1538 (4)	0.63967 (18)	0.0242 (7)	
C3	0.34336 (18)	1.1894 (4)	0.7268 (2)	0.0323 (8)	
C4	0.40930 (18)	0.9929 (5)	0.7113 (2)	0.0345 (8)	
C5	0.41077 (18)	0.9927 (5)	0.5871 (2)	0.0383 (9)	
C6	0.31483 (17)	0.9528 (5)	0.4989 (2)	0.0302 (8)	
C11	0.14981 (17)	1.1222 (4)	0.42428 (19)	0.0253 (7)	
C21	0.20048 (16)	0.9222 (4)	0.66831 (19)	0.0245 (7)	
N1	−0.01140 (18)	0.6903 (5)	0.3749 (2)	0.0320 (8)	
H1	0.26870	1.30850	0.49890	0.0290*	
H2	0.20960	1.29920	0.64640	0.0290*	
H22	0.192 (3)	0.764 (8)	0.796 (3)	0.088 (13)*	
H31	0.36540	1.35430	0.71610	0.0390*	
H32	0.34180	1.18000	0.80560	0.0390*	
H41	0.47050	1.02650	0.76520	0.0410*	
H42	0.39080	0.82920	0.72960	0.0410*	
H51	0.45100	0.86070	0.57860	0.0460*	
H52	0.43490	1.15090	0.57130	0.0460*	
H61	0.29390	0.78600	0.50860	0.0360*	
H62	0.31740	0.96480	0.42050	0.0360*	
H1A	0.016 (2)	0.832 (6)	0.363 (3)	0.057 (10)*	
H1B	0.031 (3)	0.566 (7)	0.402 (3)	0.071 (11)*	
H1C	−0.057 (2)	0.647 (5)	0.300 (2)	0.044 (8)*	
H1D	−0.038 (2)	0.728 (6)	0.436 (3)	0.072 (11)*	

### Atomic displacement parameters (Å^2^)

**Table:**

	*U*^11^	*U*^22^	*U*^33^	*U*^12^	*U*^13^	*U*^23^
O11	0.0415 (11)	0.0289 (9)	0.0191 (8)	0.0057 (8)	0.0071 (7)	−0.0059 (7)
O12	0.0338 (11)	0.0378 (10)	0.0264 (9)	0.0104 (8)	0.0093 (7)	−0.0021 (7)
O21	0.0556 (13)	0.0292 (9)	0.0254 (9)	−0.0167 (9)	0.0134 (8)	−0.0038 (7)
O22	0.0814 (16)	0.0360 (11)	0.0207 (9)	−0.0190 (11)	0.0215 (9)	−0.0004 (8)
C1	0.0336 (14)	0.0186 (11)	0.0232 (12)	−0.0030 (10)	0.0130 (10)	0.0004 (9)
C2	0.0328 (14)	0.0171 (11)	0.0227 (11)	0.0002 (10)	0.0094 (10)	−0.0009 (9)
C3	0.0398 (16)	0.0259 (13)	0.0288 (13)	−0.0043 (11)	0.0086 (11)	−0.0033 (10)
C4	0.0258 (14)	0.0362 (14)	0.0349 (14)	0.0010 (12)	0.0017 (11)	−0.0002 (11)
C5	0.0302 (15)	0.0431 (16)	0.0434 (16)	0.0057 (13)	0.0148 (12)	0.0011 (12)
C6	0.0367 (15)	0.0315 (14)	0.0260 (13)	0.0026 (11)	0.0153 (11)	0.0003 (10)
C11	0.0386 (15)	0.0173 (11)	0.0225 (12)	0.0017 (11)	0.0137 (10)	0.0037 (9)
C21	0.0301 (13)	0.0216 (11)	0.0212 (12)	0.0020 (10)	0.0081 (10)	0.0016 (9)
N1	0.0373 (14)	0.0327 (13)	0.0244 (12)	−0.0003 (11)	0.0085 (10)	0.0038 (10)

### Geometric parameters (Å, °)

**Table:**

O11—C11	1.265 (3)	C3—C4	1.521 (4)
O12—C11	1.257 (3)	C4—C5	1.520 (3)
O21—C21	1.216 (3)	C5—C6	1.525 (4)
O22—C21	1.322 (3)	C1—H1	0.9800
O22—H22	0.88 (4)	C2—H2	0.9800
N1—H1D	0.99 (3)	C3—H31	0.9700
N1—H1B	0.91 (4)	C3—H32	0.9700
N1—H1C	0.97 (2)	C4—H41	0.9700
N1—H1A	0.90 (3)	C4—H42	0.9700
C1—C11	1.519 (3)	C5—H52	0.9700
C1—C2	1.541 (3)	C5—H51	0.9700
C1—C6	1.534 (4)	C6—H61	0.9700
C2—C3	1.534 (4)	C6—H62	0.9700
C2—C21	1.523 (3)		
			
C21—O22—H22	109 (2)	C6—C1—H1	106.00
H1B—N1—H1C	112 (3)	C1—C2—H2	108.00
H1C—N1—H1D	114 (3)	C3—C2—H2	108.00
H1B—N1—H1D	108 (3)	C21—C2—H2	108.00
H1A—N1—H1D	107 (3)	H31—C3—H32	108.00
H1A—N1—H1B	110 (3)	C2—C3—H31	109.00
H1A—N1—H1C	106 (3)	C2—C3—H32	109.00
C6—C1—C11	114.70 (18)	C4—C3—H31	109.00
C2—C1—C6	111.59 (18)	C4—C3—H32	109.00
C2—C1—C11	112.6 (2)	C3—C4—H42	109.00
C1—C2—C3	111.3 (2)	C5—C4—H41	109.00
C1—C2—C21	111.10 (18)	C3—C4—H41	109.00
C3—C2—C21	111.46 (18)	H41—C4—H42	108.00
C2—C3—C4	111.90 (19)	C5—C4—H42	109.00
C3—C4—C5	111.2 (2)	C6—C5—H51	109.00
C4—C5—C6	111.2 (2)	C4—C5—H52	109.00
C1—C6—C5	112.2 (2)	C4—C5—H51	109.00
O11—C11—O12	121.2 (2)	C6—C5—H52	109.00
O11—C11—C1	119.5 (2)	H51—C5—H52	108.00
O12—C11—C1	119.25 (19)	H61—C6—H62	108.00
O21—C21—O22	122.3 (2)	C1—C6—H61	109.00
O21—C21—C2	125.2 (2)	C1—C6—H62	109.00
O22—C21—C2	112.41 (19)	C5—C6—H61	109.00
C11—C1—H1	106.00	C5—C6—H62	109.00
C2—C1—H1	106.00		
			
C6—C1—C2—C3	52.1 (2)	C1—C2—C3—C4	−54.1 (2)
C6—C1—C2—C21	−72.7 (3)	C21—C2—C3—C4	70.6 (3)
C11—C1—C2—C3	−177.29 (17)	C1—C2—C21—O21	−7.1 (4)
C11—C1—C2—C21	57.9 (2)	C1—C2—C21—O22	174.9 (2)
C2—C1—C6—C5	−53.0 (3)	C3—C2—C21—O21	−131.9 (3)
C11—C1—C6—C5	177.5 (2)	C3—C2—C21—O22	50.1 (3)
C2—C1—C11—O11	−145.4 (2)	C2—C3—C4—C5	56.3 (3)
C2—C1—C11—O12	36.0 (3)	C3—C4—C5—C6	−56.4 (3)
C6—C1—C11—O11	−16.4 (3)	C4—C5—C6—C1	55.1 (3)
C6—C1—C11—O12	165.0 (2)		

### Hydrogen-bond geometry (Å, °)

**Table:**

*D*—H···*A*	*D*—H	H···*A*	*D*···*A*	*D*—H···*A*
N1—H1A···O11	0.90 (3)	2.22 (3)	3.012 (3)	146 (3)
N1—H1A···O12	0.90 (3)	2.44 (3)	3.237 (3)	147 (3)
N1—H1B···O12^i^	0.91 (4)	1.96 (4)	2.835 (3)	161 (4)
N1—H1C···O11^ii^	0.97 (2)	1.85 (3)	2.811 (3)	168 (2)
N1—H1D···O12^iii^	0.99 (3)	1.86 (3)	2.842 (3)	174 (3)
O22—H22···O11^iv^	0.88 (4)	1.76 (4)	2.619 (3)	165 (5)
C2—H2···O21^v^	0.98	2.60	3.485 (3)	150
C3—H32···O22	0.97	2.46	2.827 (4)	102

Symmetry codes: (i) *x*, *y*−1, *z*; (ii) −*x*, *y*−1/2, −*z*+1/2; (iii) −*x*, −*y*+2, −*z*+1; (iv) *x*, −*y*+3/2, *z*+1/2; (v) *x*, *y*+1, *z*.
